# Advances in Urogenital Trauma and Reconstruction

**DOI:** 10.1155/2020/2907204

**Published:** 2020-02-19

**Authors:** Nicolaas Lumen, Francisco Martins, Enzo Palminteri, Achilles Ploumidis

**Affiliations:** ^1^Department of Urology, Ghent University Hospital, Ghent, Belgium; ^2^Centro de Cirurgia Reconstructiva Urogenital, Lisbon, Portugal; ^3^Centro di Chirurgia Uretrale e Genitale, Arezzo, Italy; ^4^Department of Urology, Athens Medical Group, Athens, Greece

Trauma is a major health burden and accounts for approximately 10% of all deaths worldwide [1]. It is the major cause of death in people aged 15–45 years [2]. Interpersonal violence and traffic road accidents are well-known and major sources of trauma, although iatrogenic injuries cannot be neglected. The urogenital tract and organs are at risk during abdomino-pelvic and perineal trauma [3, 4]. With the exception of high-grade renal trauma, urogenital trauma is rarely fatal [3]. Nevertheless, adequate initial treatment of urogenital trauma is important to reduce life-long incapacity related to renal insufficiency, lower urinary tract symptoms, urinary incontinence, or impotence [3, 4]. A specific entity of trauma is the iatrogenic injury provoked during medical procedures. Especially, the urethra is at risk for iatrogenic trauma [5]. Any trauma (iatrogenic or noniatrogenic) to the urethral mucosa can cause a subsequent urethral stricture [6]. Treatment of urethral strictures is a major challenge to the reconstructive urologist. Thorough knowledge of the anatomy of the male and female urethra, diagnostics tools, and all reconstructive options are required to obtain optimal results when treating male and female strictures. This special issue wants to provide this knowledge to the reader. In general, urethroplasty offers the best results with respect to urethral patency in the long term [7]. Nevertheless, all types of urethroplasty have an inherent risk of failure. In this case, redo-urethroplasty is an option [8]. Redo-urethroplasty might differ from primary urethroplasty and these differences need to be explored in order to provide adequate counselling to the patient with respect to outcome. Excision and primary anastomosis is one of the options to treat short bulbar and posterior strictures and provides excellent long-term results [9, 10]. This technique included mobilization of the bulbar urethra and transection at the site of the stricture and might be associated with sexual disturbances [11]. Therefore, functional outcomes and quality of life must be taken into account when performing excision and primary anastomosis. In order to reduce the surgical trauma of spongiosal transection during excision and primary anastomosis, the vessel-sparing (nontransecting) modification has been proposed [12]. Before switching to this new technique, one must be certain that this technique at least equals the results of the “older” technique. Only if there is improvement, one can proceed with a new technique.

The primary goal of cancer treatment is cure and improvement of overall survival. Penile cancer is a relatively rare cancer but with a tremendous psychological impact because the treatment will affect the genital appearance of the patient. Any attempt to maintain and/or restore the initial genital appearance as much as possible should be encouraged [13]. Glans resurfacing has been described and is currently incorporated in clinical practice guidelines for superficial penile cancer (stage ≤ T1a) [13]. Only small case series have been reported. A step-by-step description of this technique and review of the literature is needed to encourage urologists to perform this technique.

Reconstructive urologists are sometimes faced with the congenital urogenital anomalies. Reconstruction is a challenge, and the functional outcome is of utmost importance. In this issue, the laparoscopic Vecchietti operation to increase the size of the vagina in Mayer-Rokitansky-Küster-Hauser syndrome will be evaluated for functional and sexual outcomes.

*Nicolaas Lumen* *Francisco Martins* *Enzo Palminteri* *Achilles Ploumidis*
